# Dealing with research participants’ complaints in HIV/AIDS prevention studies: experiences from Zimbabwe

**DOI:** 10.1186/1742-4690-9-S2-P227

**Published:** 2012-09-13

**Authors:** R Gunda, S Ruzario, R Chekera, M Phiri, R Gutsire

**Affiliations:** 1 Medical Research Council of Zimbabwe, Harare, Zimbabwe

## Background

The Medical Research Council of Zimbabwe (MRCZ) houses the National Ethics Committee which safeguards the rights, safety and well-being of all research participants. Research participants in AIDS Prevention Trials, including HIV/AIDS vaccine trials, do from time to time have complaints during research implementation. The National Ethics Committee takes these complaints seriously and works tirelessly to address all complaints that research participants report to them in order to continuously improve the ethical conduct of AIDS research. The research community as a whole suffers when even a few investigators ignore the basic principles of ethics.

## Methods

We ensured that all research participants in Zimbabwe are well informed of the National Ethics Committee`s complaint procedure. Adequate information on how to lodge a complaint is supplied upon request. Complaints are listed with all relevant details and are investigated fully by the Council. Depending on each complaint, urgent meetings with investigators and study staff are done. Urgent for-cause site inspections, interviews with study participants, impromptu meetings with Community Advisory Board members and the communities are also done. Community Research Ethics Awareness Outreaches are conducted periodically and annual Research Ethics Forums are held for researchers and participants to bridge the gap between them.

## Results

The MRCZ managed to address all participants complaints reported in 2011. The number of for-cause site inspections for HIV/AIDS Prevention Trials increased from 2 in 2010 to 8 in 2011. Complaints included participants spending longer times at a research site than the time written on the informed consent, insufficient food given at research site, laboratory results not being issued on time as promised, confidentiality not being maintained and insufficient bus fare reimbursements being given.

## Conclusion

A functional system to handle complaints from research participants helps to improve the ethical conduct of research. Experiences from the Medical Research Council of Zimbabwe will be shared.

